# Supratentorial Sporadic Hemangioblastoma: A Case Report With Mutation Profiling Using Next-Generation DNA Sequencing

**DOI:** 10.7759/cureus.39818

**Published:** 2023-06-01

**Authors:** Mohiuddin M Taher, Najwa A Bantan, Mustafa H Alwalily, Muhammad Saeed, Nuha M Taher, Meriem Bouzidi, Raid A Jastania, Kamal B Balkhoyour

**Affiliations:** 1 Science and Technology Unit, Deanship of Scientific Research, Umm Al-Qura University, Makkah, SAU; 2 Department of Medical Genetics, Umm Al-Qura University College of Medicine, Makkah, SAU; 3 Department of Radiology, Al-Noor Specialty Hospital, Makkah, SAU; 4 Department of Neurosurgery, Al-Noor Specialty Hospital, Makkah, SAU; 5 Department of Laboratory Medicine, Division of Histopathology, Al-Noor Specialty Hospital, Makkah, SAU; 6 Department of Pathology, Umm Al-Qura University College of Medicine, Makkah, SAU

**Keywords:** next-generation dna sequencing, brain tumor, hemangioblastoma, vhl-related syndrome, ngs, supratentorial lesions, ion torrent, ion proton, intracranial tumors, targeted therapy

## Abstract

The present study aimed to determine genomic changes in sporadic intracranial hemangioblastoma (HBL), and the mutation patterns were analyzed using next-generation DNA sequencing (NGS). In this NGS analysis of the HBL tumor, 67 variants of 41 genes were identified. Of these, 64 were single-nucleotide variants (SNVs), two were exonic insertions and deletions (INDEL), and one was an intronic INDEL. In total, 15 were missense exonic variants, including an insertion variant in the *NRAS *gene, c.1_2insA, and a deletion variant, c.745delT, in the *HNF1A *gene, both of these mutations produced a termination codon. Other exonic missense variants found in the tumor were *CTNNB1*, *FGFR3*, *KDR*, *SMO*, *HRAS*, *RAI1*, and a *TP53 *variant (c.430C>G). Moreover, the results of the present study revealed a novel variant, c.430C>G, in *TP53 *and two missense variants of *SND1 *(c.1810G>C and c.1814G>C), which were also novel. *ALK *(rs760315884) and *FGFR2 *(rs1042522) missense variants were reported previously. Notably, a total of 10 previously reported single-nucleotide polymorphisms (SNPs) were found in this tumor in genes including *MLH1 *(rs769364808), *FGFR3 *(rs769364808), two variants (rs1873778 and rs2228230) in *PDGFRA*, *KIT *(rs55986963), *APC *(rs41115), and *RET *(rs1800861). The results of this study revealed a synonymous mutation (SNP) in c.1104 G>T; p. (Ser368Ser) in the *MLH1 *gene. In this amino acid (AA) codon, two other variants are also known to cause missense substitutions, c.1103C>G; p. (Ser368Trp); COSM6986674) and c.1103C>T; p.(Ser368Leu; COSM3915870), were found in hematopoietic and urinary tract tissue, respectively. However, three SNPs found in genes such as *ALK*, *KDR*, and *ABL1* in the HBL tumor in this study were not reported in UCSC, COSMIC, and ClinVar databases. Additionally, 19 intronic variants were identified in this tumor. One intronic SNV was present in each of the following genes: *EGFR*, *ERBB4*, *KDR*, *SMO*, *CDKN2B*, *PTEN*, *PTPN11*, *RB1*, *AKT1*, and *ERBB2*. In *PIK3CA *and *FBXL18 *genes, two intronic variants were present, and in the *SND1 *gene, three intronic variants were detected in the HBL tumor presented in this study. Notably, only one of these was reported in the catalog of somatic mutations in cancer. Only one 3’-untranslated region (UTR) insertion variant in the *NRAS *gene (c.*2010T>AT) was detected in the tumor of the present study, and this was a splice site acceptor. A *TP53 *intronic mutation (c.782+1G>T) was the only pathogenic splice_donor_variant found in this HBL tumor. The frequency of variants and Phred scores were markedly high, and the p-values were significant for all of the aforementioned mutations. In summary, a total of 15 missense, 10 synonymous, and 19 intronic variants were identified in the HBL tumor. Results of the present study detected one novel insertion in *NRAS *and one novel deletion in *HNF1A *genes, a novel missense variant in the *TP53 *gene, and two novel missense variants of *SND1*. Hotspot mutations in other cancer driver genes, such as *PTEN*, *ATM*, *SMAD4*, *SMARCB1*, *STK11*, *NPM1*, *CDKN2A*, and *EGFR*, which are frequently affected in gliomas, were not found in the tumor of the present study. Future studies should aim to validate oncogenic mutations that may act as novel targets for the treatment of these tumors.

## Introduction

Hemangioblastoma (HBL) is a slow-growing vascular tumor that is often found in the brain’s posterior fossa (brain stem and cerebellum), but, occasionally, is seen in the retina, pituitary gland, and spinal cord [1,2]. These tumors contain networks of small blood vessels interspersed with bland-looking, lipid-loaded stromal cells. They are classified as benign World Health Organization (WHO) Grade I of uncertain histogenesis. The stromal cells of this tumor appear dull under a microscope, with substantial nuclear pleomorphism, mimicking some type of carcinoma [3,4]. However, when they grow in size intracranially, these tumors may cause neurological symptoms, such as headaches, weakness, sensory loss, ataxia, nausea, vomiting, dizziness, nystagmus, and hydrocephalus. In primary brain tumors, HBLs account for 1-2%, and in primary posterior fossa tumors, they account for 7 to 12% [5]. The majority of HBLs occur sporadically without a known cause in young or middle-aged adults and often grow as a single tumor. The mean age at presentation is 47 years for sporadic and 29 years for von Hippel-Lindau (VHL) syndrome HBLs, with a 1-1.25:1 ratio of males to females [1,6,7]. However, several patients may develop HBLs known as VHL syndrome (OMIM #193300) due to germline inactivating mutations in the *VHL *tumor suppressor gene (NM_000551.3, 3p25-26), which exhibits an autosomal dominant inheritance pattern. Throughout their lifetime, these patients usually develop multiple tumors within the brain and spinal cord [8]. Notably, VHL tumors possess the ability to grow in other places as well, viz., liver, pancreas, lung, adrenal glands, kidney, and urinary bladder [9]. Around, 20-25% of HBL cases are caused by VHL. Both sporadic and VHL-associated HBLs develop predominantly in the cerebellum; however, VHL-related HBLs are exclusively supratentorial [6,10].

Results of previous studies demonstrated recurrent chromosomal copy number losses and few chromosomal gains in capillary HBL using droplet digital polymerase chain reaction (PCR) and comparative genomic hybridization (CGH) arrays [11,12]. Analysis of CGH and next-generation DNA sequencing (NGS) results of sporadic cerebellar and spinal HBLs demonstrated the DNA copy number losses of chromosome 3, and the loss of heterozygosity and/or deletion of VHL were the most common abnormalities detected [12,13]. In addition, losses of chromosomes 6, 9, 11, and 18q and a gain of chromosomes 1, 7, and 19 were also determined [13-16]. Moreover, Mehrian-Shai et al. identified 23 candidate genes and microRNAs (mir), such as has-mir-196a-2, to determine HBL pathogenesis [11]. Taibe et al. demonstrated the positive expression of VEGFR2/CA9/Glut1 and HAF (SART1800; squamous cell carcinoma antigen recognized by T cells), and the presence of *HIF2A *mutations in sporadic CNS-HBL [17]. Results of a previous study reported the overexpression of *EGFR *and *TGF-α* in VHL-related CNS-HBLs using real-time PCR [16].

At present, NGS is recognized as a powerful tool for the analysis of single-nucleotide variants (SNVs), copy number variations (CNVs), gene fusions, and epigenetic alterations, as well as in determining the functional impact of genomic alterations in numerous types of cancers. NGS is also used for the identification of additional somatic genetic variations that may aid in tumor classification, the development of targeted therapy, and the determination of the heritability of certain cancers [18]. NGS is sensitive and effective and is commonly utilized in numerous molecular diagnostic laboratories. The *VHL *mutation in HBL tumors that could not be detected using Sanger sequencing was successfully detected using NGS, as demonstrated by Coppin et al. [19]. At present, few studies have focused on mutation profiling in spinal and intracranial HBL using NGS methodology. Numerous previous studies focused on NGS of brain tumors did not include HBL tumors due to their rarity. Thus, this study aimed to determine the mutation profile of a sporadic HBL tumor through NGS on the IonProton NGS system. The results of this study provide an evaluation of mutational signatures of a Grade I HBL tumor.

## Case presentation

Patient demographics

A 32-year-old female Saudi patient with an intellectual disability was admitted to the emergency room in a postictal state following a generalized epileptic fit with dense right-sided hemiparesis and was admitted with a provisional diagnosis of intractable epilepsy. She had a history of focal fits for three years involving the right side of the body, which became generalized with tonic-clonic seizures over the three months before admission. The fits were intractable, could not be controlled using carbamazepine, and were associated with chronic mild right-sided hemiparesis. The patient had a past medical history of biliary stenting and cholecystectomy for common bile duct stones and uterine fibroid. There were no other known associated diseases. On examination, the patient was conscious with mental retardation and dense right-sided hemiparesis. The final diagnosis was made following radiological, histopathological, and immunological examinations.

Histopathological and immunological examination

Details of histopathological and immunological methods were published by us previously [20,21]. The formalin-fixed paraffin-embedded (FFPE) tumor tissue used in this NGS analysis was obtained from the Al-Noor Specialty Hospital, Makkah. Sections (thickness, 4 μm) were prepared on Citoglas adhesion microscope slides (Citotest) and routinely stained using hematoxylin and eosin (H and E) on a Dako Coverstainer (Agilent Technologies, Inc.) and immunohistochemistry Ventana BenchMark XT automated Stainer, Ventana Medical Systems, Inc.). After staining, the images were processed using NIKON Digital Microscope Camera DS-Ri1, with image software NIS Elements v.4.0 [22,23]. The tumor was classified based on similarity to the constituent cells of the central nervous system (CNS), according to the WHO Grading system [4].

**Figure 1:**
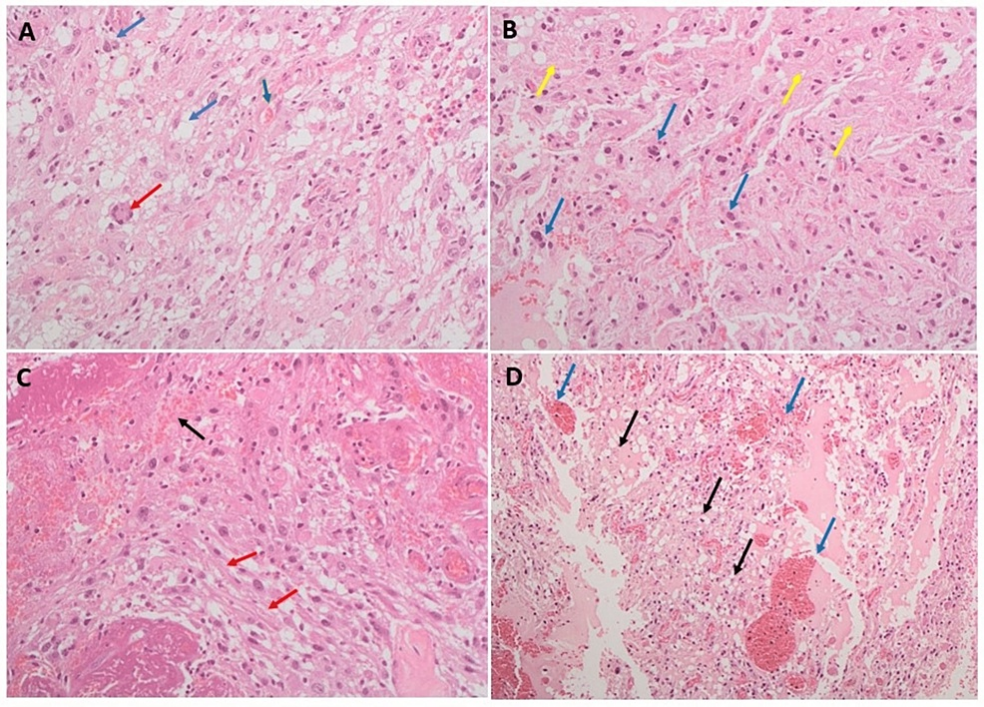
Hematoxylin and eosin-stained microscopic images of hemangioblastoma tumor. (A) The tumor cells comprise benign lipid-laden foamy stromal cells surrounding the network of capillaries. The blue arrow shows lipid-laden foamy stromal cells, the red arrow shows lipid-laden foamy giant cells, and the green arrow shows small capillaries surrounded by interstitial lipid-laden tumor cells. (B) The tumor tissue depicts nuclear atypia within the stromal cells (blue arrows) and large areas of hyalinization (yellow arrow). (C) Tumor tissue with hyalinized stroma containing (red arrow) and with large branching vessels (black arrow). (D) Classic hemangioblastoma features depicting numerous vessels (blue arrow) and vacuolated stromal cells (black arrow).

The H and E staining of the tumor revealed classical HBL features depicting numerous large branching vessels, vacuolated stromal cells exhibiting nuclear atypia, and large areas of hyalinization. Representative H and E images are displayed in Figure 1. As shown in the figure, tumor cells comprised benign lipid-laden foamy stromal cells surrounding the network capillaries (Figure 1A). Tumor tissue depicted nuclear atypia within the stromal cells and large areas of hyalinization (Figure 1B). Tumor tissue also contained hyalinized stroma with large branching vessels (Figure 1C). The classical HBL features, including numerous vessels and vacuolated stromal cells, are also clear in Figure 1D.

**Figure 2:**
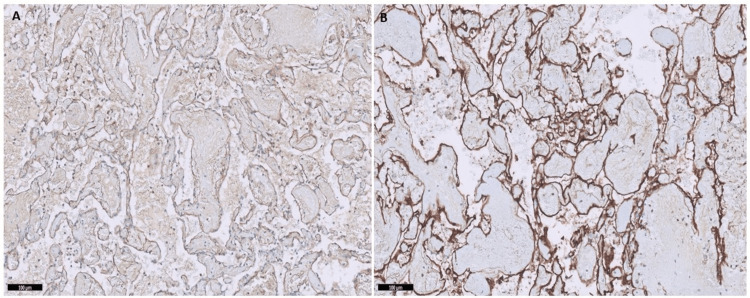
Immunohistochemistry of hemangioblastoma tumor. Immunostaining of the hemangioblastoma tumor revealed positivity for CD31 (A) and CD34 (B) antibodies.

**Figure 3:**
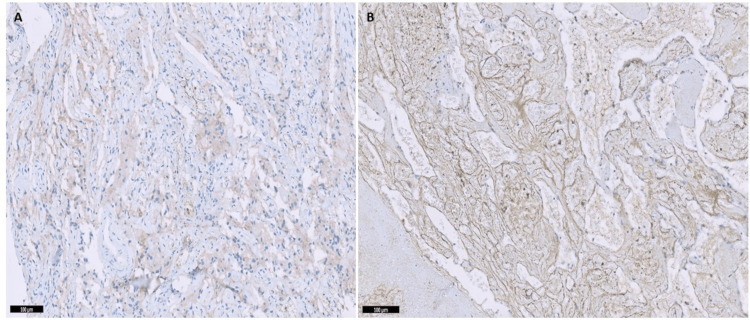
Immunohistochemistry of hemangioblastoma tumor. Immunostaining of the hemangioblastoma tumor showed weak positivity with CD56 antibody panel (A) and inhibin antibody panel (B).

Immunohistochemical results revealed positive CD31 and CD34 staining of the vessel walls of the HBL tumor, specifically in the endothelial cells (Figure 2), revealing the highly vascular nature of this hemangioblastoma tumor surrounding the neoplastic stromal cells. In addition, the staining of stromal cells was weak for CD56 and inhibin (Figure 3).

Radiographic features

**Figure 4:**
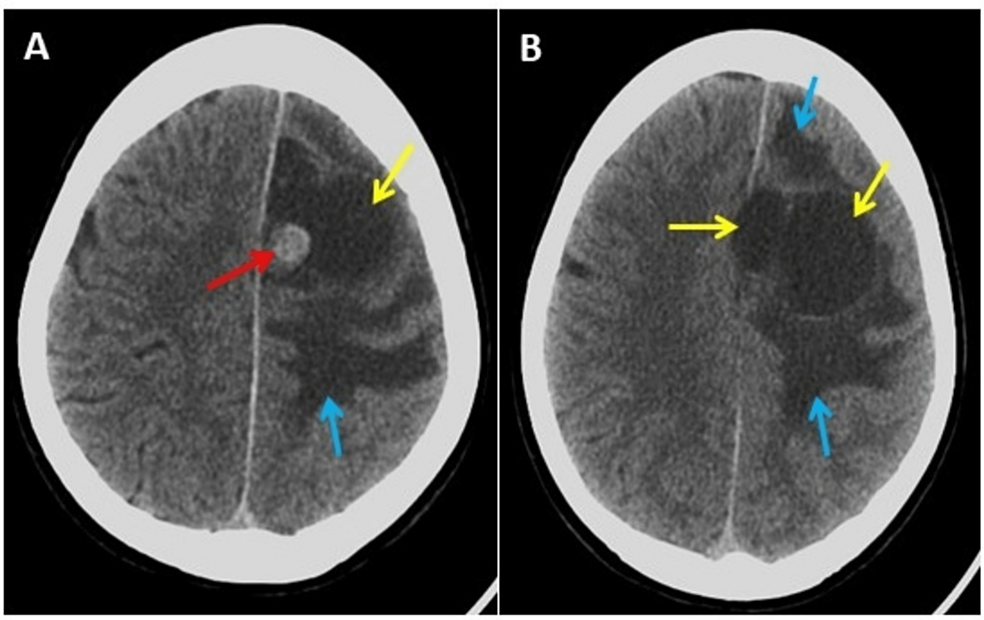
Brain computed tomography scan without contrast. Axial (A) and (B) lower level showing a left frontal multicystic lesion (yellow arrows) with a small rounded hyperdense nodule (red arrow) and surrounded with massive edema (blue arrows).

An emergency brain computed tomography (CT) scan was performed without contrast (Figure 4), and the results demonstrated a well-defined, left frontal parasagittal, intra-axial multicystic lesion with a hyperdense nodule of 40 Hounsfield units (HU). This was surrounded by edema, causing effacement of the sulci and compression of the lateral ventricle.

**Figure 5:**
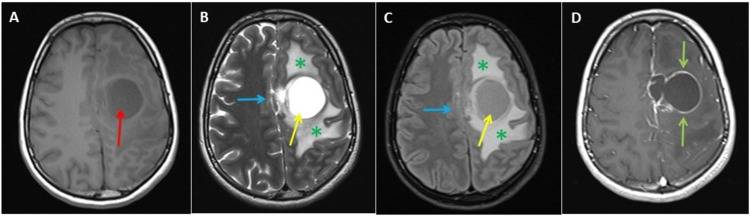
Magnetic resonance imaging (MRI) of the brain showing the cystic lesion and post-contrast images of the cystic lesion. MRI of the brain shows a large left frontal coalescent cystic lesion abutting the interhemispheric fissure. The cystic fluid was of mild T1 hyperintensity (panels A, red arrow), and transverse relaxation time/fluid-attenuated inversion recovery hyperintensity relative to the cerebrospinal fluid (panels B and C, yellow arrows). This lesion displays marked perilesional edema (panels B and C, green asterisks) and mass effect (panels B and C, blue arrows). Post-contrast T1 images display peripheral ring-like enhancement of the cyst wall (panel D, green arrows).

Multi-sequential, multi-planar, pre-, and post-gadolinium magnetic resonance imaging (MRI) examination of the brain was performed on Siemens 3T MAGNETOM Skyra MRI scanner. High-quality images were processed at low-dose performance on the Volara™ digital data acquisition system. The MRI investigations revealed a large left frontal coalescent cystic lesion abutting the interhemispheric fissure and compressing the superior sagittal sinus. It measured 4.6 × 4.2 × 3.5 cm. The cystic fluid was of mild longitudinal relaxation time (T1) hyperintensity, and transverse relaxation time/fluid-attenuated inversion recovery (T2/FLAIR) hyperintensity relative to cerebrospinal fluid (CSF), as shown in Figure 5.

**Figure 6:**
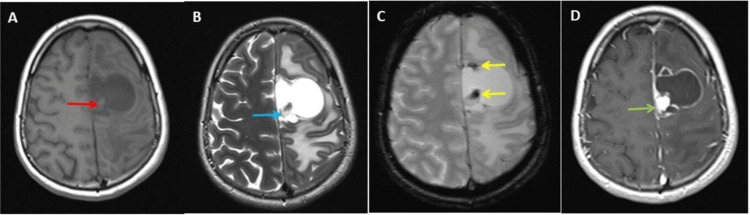
Magnetic resonance imaging (MRI) of the brain at a higher level showing the cyst and the hemoglobin (HEMO)-sensitive image of the cystic lesion and post-contrast images. The MRI of the brain at a higher level shows a small eccentric mural nodule in the cyst abutting the pia matter. This mural nodule isointense to white matter on T1-weighted image (panel A, red arrow), displays high T2 signals (panel B, blue arrow), and shows blooming/signal drop on the HEMO-sensitive image at the tip of the nodule and wall of the cyst (panel C, yellow arrows). Post-contrast T1 images display avid enhancement of the mural nodule abutting pia matter (panel D, green arrows).

It exhibited a rounded solid component medially abutting the pial layer, measuring 1.5 cm in diameter of T1 isointensity, T2/FLAIR hyperintensity relative to the gray matter, restricted diffusion on diffusion-weighted imaging (DWI), and peripheral hypointensity on T2W and hemoglobin derivatives (HEMO), suggestive of subacute hemorrhage (Figure 6).

**Figure 7:**
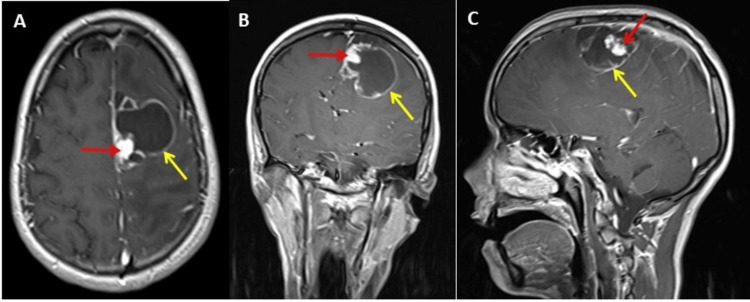
Post-contrast axial, coronal, and para-sagittal magnetic resonance imaging (MRI) of the brain. MRI brain post-contrast images axial (A), coronal (B), and para-sagittal (C); T1 images of the brain showing mural nodule abutting the pia matter and displaying avid enhancement (red arrows). Also, peripheral ring enhancement of the cystic lesion is evident (yellow arrows).

Post-contrast T1 images demonstrated cyst wall enhancement and homogeneous avid enhancement of the mural nodule (Figure 7). In addition, the results of this revealed a subfalcine herniation, midline shift by 7 mm to the right side, and compression of the left lateral and third ventricles. Both optic nerves were tortuous with distensions of the nerve sheaths. Posterior flattening of the left eye globe was also noted. The right globe demonstrated posterior herniation of the vitreous fluid (not shown in the figure).

Treatment and prognosis

The patient had undergone a tumor excision performed using occipital craniotomy. Under general anesthesia in the supine position with the head fixed with the Mayfield, a left frontal parasagittal osteoplastic craniotomy bone flap was created. The dura opened square-shaped and was based on the superior sagittal sinus. The tumor was found to be cystic and surfacing at a small parasagittal area. A proper line of cleavage was found between the thin-walled cystic tumor and the brain tongue, covering the area near the pointing part of the tumor. The rest of the tumor was embedded within the brain tissue with the challenge of dissection between the thin wall and the healthy brain tissue. The cystic element was aspirated with the brain cannula. The aspirate was clear and xanthochromic. Trials of deep dissection failed and the thin-walled cyst opened. The free thin wall was removed for histopathological examination. The surgical microscope was used to explore the cavity, and a solid mural nodule-like section was found attached to the brain tissue at the falcine side, which was surrounded by small fine trabeculae. The trabeculae were broken with the aid of both bipolar coagulation and the arachnoid knife, and the nodule was microscopically resected utilizing the bipolar coagulation around the nidus. Finally, hemostasis was secured without violation of the healthy brain tissue. On discharge, the patient was conscious with moderate right-sided hemiparesis.

**Figure 8:**
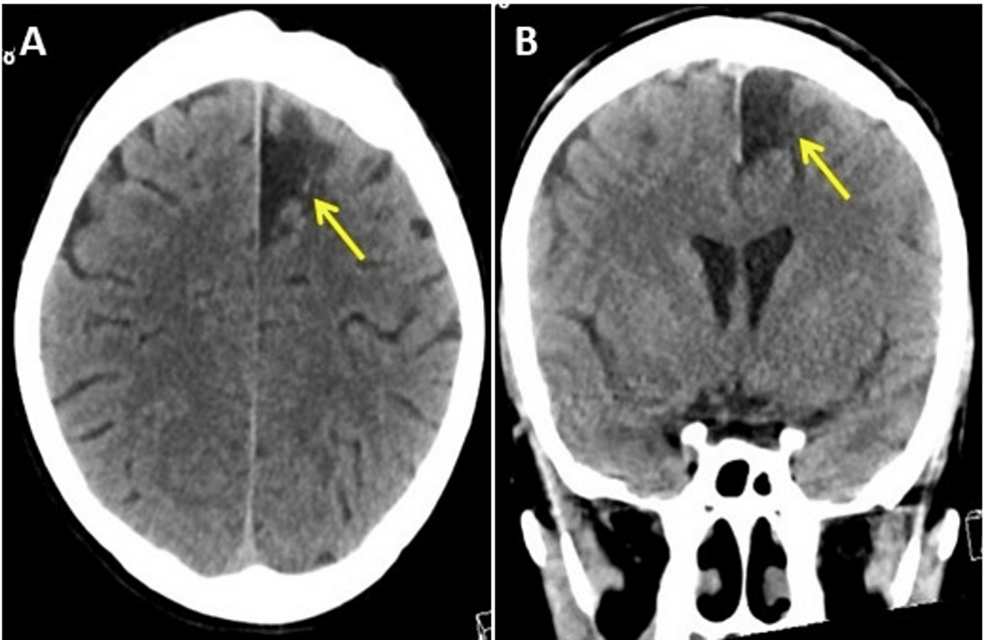
Follow-up computed tomography (CT) scan images of the brain two years after the surgery. Follow-up CT scan two years after surgery, panel (A) axial view, and panel (B) coronal view of the brain showing focal cortical volume loss and encephalomalacia (yellow arrows) at the surgical bed with complete resolution of the edema and mass effect.

After two years of follow-ups in the outpatient department, the patient was fully conscious but developmentally challenged with mild right-sided hemiparesis. Epileptic fits were controlled using phenytoin and carbamazepine. The patient experienced a few headaches and was living a healthy life. Follow-up CT scans revealed focal cortical volume loss and encephalomalacia at the surgical bed, with complete resolution of the edema and mass effect (Figure 8). Complete surgical resection of HBL is curative and exhibits low morbidity and only 2% mortality following surgery [6].

Next-generation DNA sequencing analysis

DNA isolation was performed using the QIAamp DNA FFPE kit (cat. no. 56404; Qiagen GmbH). As described previously [20], DNA (10 ng) was sequenced using the Ion PI v3 Chip kit (cat no. A25771) with the Ion Proton system (Thermo Fisher Scientific, Inc.) [21-23]. IonExpress Barcode Adapters 1-16 (Cat. No. 4471250) tagged libraries were prepared using Ion AmpliSeq™ Cancer panel v1 primer pool (cat no. 4471262) which generates 190 amplicons in a single reaction (Table 1).

**Table 1:** List of 46 genes present in the Ion AmpliSeq cancer panel v1 used in this next-generation DNA sequencing analysis of the hemangioblastoma tumor.

Gene symbol	Genome location	Gene symbol	Genome location	Gene symbol	Genome location
*ABL1*	9:130713946-130885683	*FGFR2*	10:121479857-121598403	*NOTCH1*	9:136494433-136545786
*AKT1*	14:104769349-104794124	*FGFR3*	4:1793312-1808872	*NPM1*	5:171387648-171411137
*ALK*	2:29192774-29921566	*FLT3*	13:28003274-28100592	*NRAS*	1:114704469-114716894
*APC*	5:112737888-112846239	*GNAS*	20:58852714-58911192	*PDGFRA*	4:54229097-54298247
*ATM*	11:108222832-108369099	*HNF1A*	12:120978749-121001269	*PIK3CA*	3:179148523-179240093
*B-Raf*	7:140730665-140924928	*HRAS*	11:532243-535550	*PTPN11*	12:112419112-112504764
*CDH1*	16:64943753-65122198	*IDH1*	2:208236265-208254322	*PTEN*	9:95442980-95508661
*CDKN2A*	9:21968056-21974866	*JAK1*	1:64833229-64966504	*RB1*	13:48303726-48481890
*CSF1R1*	5:150053291-150113372	*JAK3*	19:17824786-17848032	*RET*	10:43077027-43130351
*CTNNB1*	3:41220551-41239888	*KDR*	4:55078259-55125595	*SMAD4*	18:51030213-51085039
*EGFR*	7:55019101-55211628	*KIT*	4:54657918-54740715	*SMARCB1*	22:23786963-23834505
*ERBB2*	17:39700080-39728662	*KRAS*	12:25209431-25250803	*SMO*	7:129188872-129213545
*ERBB4*	2:211375717-212538841	*MET*	7:116672405-116796342	*STK11*	19:1206464-1228435
*FBXW7*	4:152320544-152536063	*MLH1*	3:36993332-37050918	*TP53*	17:7668402-7687538
*FGFR1*	8:38411138-38467845	*MPL*	1:43337849-43352772	*VHL*	3:10141008-10152220

**Figure 9:**
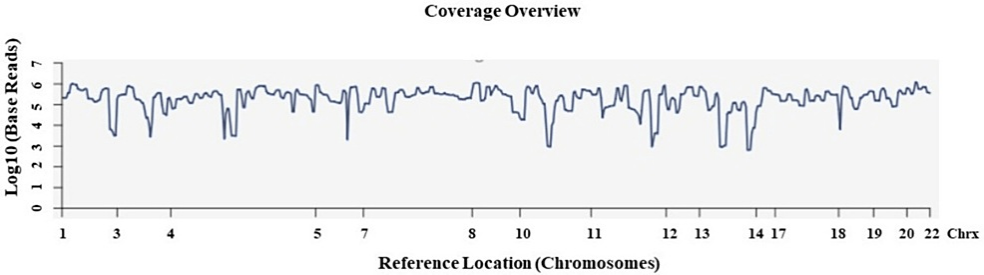
Sequences alignment analysis of the target regions and sample coverage overview. Sequences alignment of the target regions (CP.20131001.designed) to the reference genome (Hg 19), and sample coverage analysis was performed by the Ion Torrent Suite software v 5.0.2. Chrx = chromosome; CP = cancer panel; Hg = human genome

Sequencing was carried out using the Ion PI Hi-Q Sequencing 200 kit (cat no. A26433). Following sequencing, amplicon sequences were aligned to the human reference genome (Hg) 19, (GRCh37), in the target region of the cancer panel (CP) using the Torrent Suite software v.5.0.2 (Thermo Fisher Scientific, Inc.) [24] (Figure 9). NGS analysis revealed 2,983,520 mapped reads, the on-target reads were 95.08%, and the mean depth and uniformity of base coverage were 13,747 and 82.02%, respectively.

The routine practice is to align the produced sequence reads to the reference genome (GRCh37/hg19) sequence, which is widely used for NGS analyses and genome annotations [25]. Although it is a very useful resource, there are some drawbacks in GRCh37, and several of its characteristics are not ideal for application to NGS analyses, particularly for some populations [26], such as ethnic ancestries being under-represented due to its bias toward European and African ancestries. These issues can also complicate short-read mapping and variant callings. Several studies have suggested approaches to overcome these problems. For example, by constructing reference genomes specific to ethnic populations of interest, or by the addition of sequences not present in the reference genome, such as by substituting its minor variants with the major variants from African, Asian, or European populations [27].

**Figure 10:**
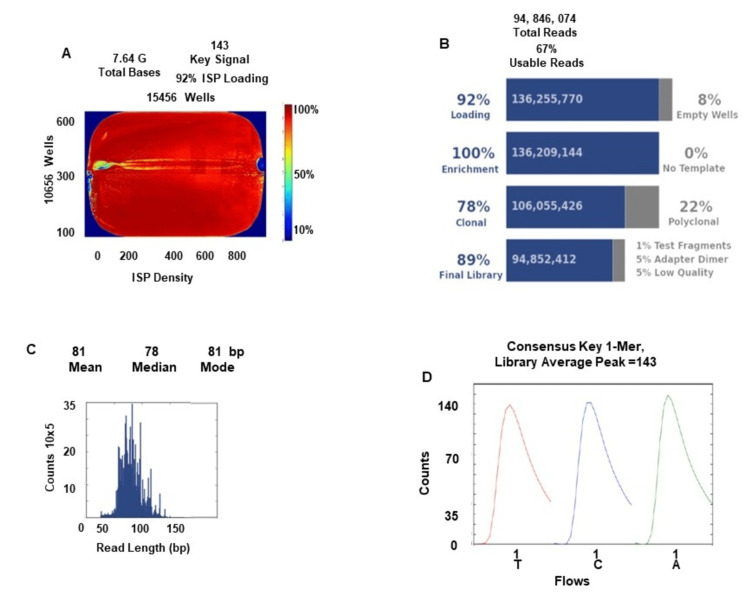
Summary of the Chip sequencing metrics of the hemangioblastoma tumor on Ion Proton. (A) The chip has loaded well by visualizing quite clearly in the bead density diagram, as well as ISP summary details such as loading (%). (B) Enrichment, clonality, and final library (%). (C) Read length. (D) The key incorporation graph shows the average signal readings for flows of the bases T, C, and A in the library key.

**Figure 11:**
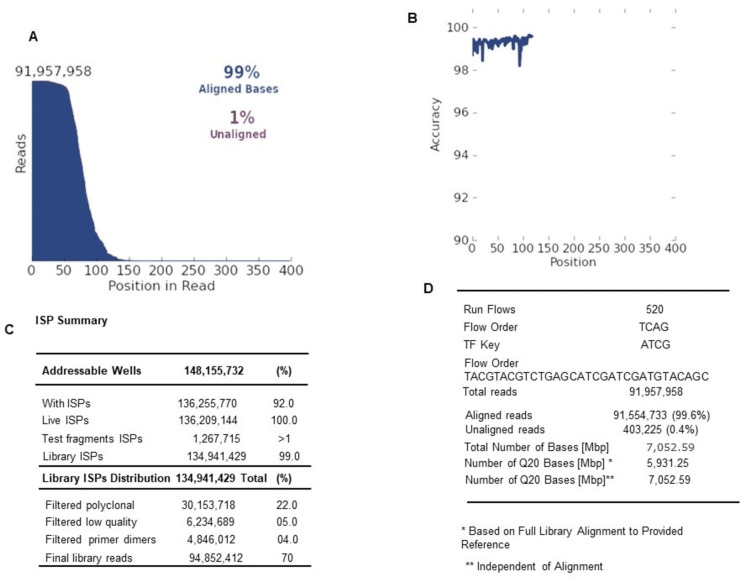
Chip sequencing metrics of the hemangioblastoma tumor on Ion Proton. (A) Aligned and unaligned reads. (B) Mean raw accuracy (1×) 99.3. (C) ISP summary details, such as the number of wells with library ISPs, test fragments ISPs, and library ISP distribution. (D) Chip run metrics such as the total flows, flow order, number of total reads, number of aligned reads and unaligned reads, and number of Q20 bases.

The quality assessment metrics of this Chip run generated by Torrent Suite software are shown in the Appendices (Figures 10, 11). We have shown the bead loading, the ISP density image is a pseudo-color image of the Ion Chip plate showing percent loading across the physical surface. The percentage of chip wells that contain a live ISP was 92% and for empty wells was 8%. Summary of aligned and unaligned reads, total bases, key signal, total reads, usable reads, ISP summary details such as loading percentage, enrichment which was 100%, clonality (polyclonal 22%, clonal 78%); the final library was 89%, adapter dimers were 5%, test fragments were 1%, and low-quality library reads were 5%. The read length histogram shows the trimmed lengths of all called reads also presented (Appendices, Figure 10).

For the tumor in this study, out of 190 amplicons, multiplexed PCR produced 189 amplicons in a single tube (Table 2). Target read depth (or coverage) is the number of independent reads with overlapping alignment at a locus of interest, i.e., the percent of all bases covered by reads aligned to the reference that covered bases in target regions. The target base coverage at 100× and 500× was 96.84% and 92.96%, respectively (Table 2). In this sequence, amplicons with 100 reads and 500 reads were 97.35% and 92.59%, respectively. This high level of accuracy and the high depth of coverage allows us to reliably detect low-frequency mutations with high confidence.

**Table 2:** Sequencing coverage analysis report generated by Torrent Suite software v.5.0.2 for the hemangioblastoma tumor.

Target base coverage		Amplicon read coverage	
Bases in target regions	13,311	Number of amplicons	189
Percent base reads on target	83.47%	Percent assigned amplicon reads	95.08%
Average base coverage depth	13,747	Average reads per amplicon	15,009
Uniformity of base coverage	82.02%	Uniformity of amplicon coverage	80.42%
Target base coverage at 1×	100%	Amplicons with at least 1 read	100%
Target base coverage at 20×	100%	Amplicons with at least 20 reads	100%
Target base coverage at 100×	96.84%	Amplicons with at least 100 reads	97.35%
Target base coverage at 500×	92.96%	Amplicons with at least 500 reads	92.59%
Target bases with no strand bias	97.12%	Amplicons with no strand bias	97.88%
Percent end-to-end reads	93.98%	Amplicons reading end-to-end	97.35%

Variant call format (VCF) files were generated by running the Torrent Variant Caller Plugin v5.2. for further mutation identification. The VCF file data were analyzed using Ion Reporter v5.18.4.0 (Thermo Fisher Scientific, Inc.), which calculated allele coverage, allele frequency, allele ratio, variant impact, clinical significance, Polymorphism Phenotyping v2 (PolyPhen-2) score, Phred score, Sorting Intolerant from Tolerant (SIFT) score, Grantham score, and Functional Analysis Through Hidden Markov Models (FATHMM) score [22,28]. The PolyPhen-2 score predicts the potential impact of substitution in the variant on the structural and functional aspects of the protein. True mutations were considered based on a Phred quality score >20 and mutations with a p‑value <0.05 were considered significant. Variants that do not fall into this criterion were filtered out [22,28].

## Discussion

HBL tumors are rarely detected in the supratentorial region of the brain and are often infratentorial tumors. As of November 2022, there were only 162 known cases of supratentorial HBL; 68 were reported to be without VHL syndrome [7]. HBL tumors develop sporadically in 67% of all cases, and in 33% of cases, as a feature of VHL syndrome [1,2]. In addition, among supratentorial HBL tumors, 60% were diagnosed with VHL disease, and 40% were non-VHL tumors [2]. In the case of supratentorial HBL, the average age reported is 36 years, and the male-to-female ratio is 1.3:1 [6]. As reported previously, supratentorial HBLs are most commonly located in the frontal lobe, followed by the parietal and temporal lobes [6]. In the present case, the HBL tumor was located in the left frontal lobe. Typically, HBL tumors are composed of a well-demarcated enhancing mass with a cyst and a mural nodule that avidly enhances and often has prominent serpentine flow voids. Histological features of HBL tumors include sheets of polygonal cells and an abundance of arborizing capillary networks, with tumor cells comprising eosinophilic cytoplasm, and several extracellular and intracytoplasmic hyaline globules. The tumor characteristics in the present case are in accordance with the literature [6,7]. Histopathological investigations confirmed that the tumor was HBL with common characteristics and immunostaining of inhibin alpha [29], and endothelial cell markers, CD31, CD34, and CD56 [30], also confirmed the present case as HBL.

Initial analysis using Ion Reporter software demonstrated that 67 variants of 41 genes passed all filters. Of that, 64 were SNVs, with one exonic insertion, one exonic deletion, and one intronic insertion. A summary of all missense and exonic insertion and deletion (INDEL) mutations found in the HBL tumor is displayed in Table 3.

**Table 3:** Missense and INDEL mutations found in the hemangioblastoma tumor. cDNA = coding DNA; AA = amino acid; PolyPhen = polymorphism phenotyping; INDEL = insertion and deletion

Gene name	Genotype	Reference	Observed allele	Allele frequency (%)	cDNA changed	AA changed	Exon	PolyPhen
NRAS	AT/AT	A	AT	94.96	c.1_2insA	p. (Met1fsX1)	2	
ALK	C/C	A	C	100	c.3750T>G	p. (Ile1250Met)	25	1
CTNNB1	T/T	G	T	97.1	c.149G>T	p. (Gly50Val)	3	0.725
PIK3CA	G/A	G	A	16.5	c.2119G>A	p. (Glu707Lys)	14	0.998
FGFR3	A/T	A	T	41.67	c.1246A>T	p. (Ile416Phe)	9	0.282
KDR	A/G	A	G	79.07	c.862T>C	p. (Phe288Leu)	7	0.014
SND1	C/C	G	C	100	c.1810G>C	p. (Ala604Pro)	17	0.996
SND1	C/C	G	C	100	c.1814G>C	p. (Gly605Ala)	17	0.993
SMO	G/G	T	G	97.14	c.540T>G	p. (Asn180Lys)	3	0.997
FGFR2	C/C	A	C	95.35	c.872T>G	p. (Ile291Ser)	7	0.053
HRAS	A/T	A	T	30.19	c.2T>A	p. (Met1Lys)	2	0.991
HNF1A	AT/A	AT	A	62.5	c.745delT	p. (Ser249HisfsX80)	4	
TP53	C/A	C	A	83.33	c.432G>T	p. (Gln144His)	5	0.999
TP53	G/C	G	C	90.91	c.430C>G	p. (Gln144Glu)	5	0.727
RAI1	A/A	G	A	100	c.2713G>A	p. (Glu905Lys)	3	0.489

In the tumor of the present study, 15 missense substitutions were detected, including one insertion and one deletion mutation in 13 genes. Among these 15 variants, eight were homozygous and seven were heterozygous. In exon 2 of the *NRAS *gene, an insertion (c.1_2insA) caused a frameshift mutation p.(Met1fsX1), producing a termination codon in place of the start codon. Notably, this mutation had not been previously reported; thus, this is a novel mutation in these genes. In exon 4 of the *HNF1A *gene, a deletion was found (c.745delT) causing a frameshift p. (Ser249HisfsX80). This mutation was also novel; however, in this amino acid (AA) codon, another (COSM7239830) terminating c.746C>G; p. (Ser249*) mutation was reported in International Cancer Genome Consortium (ICGC) project Lung Cancer-KR in one sample of squamous cell carcinoma of the lung.

All mutations were verified using various databases, viz, Catalogue of Somatic Mutations In Cancer (COSMIC), Exome Aggregation Consortium (ExAC), and Single Nucleotide Polymorphism Database (dbSNP) to further confirm whether the variants found in the NGS analysis were novel or had been previously reported. Missense variants found in *NRAS*, *CSDE1*, *CTNNB1*, *FGFR3*, *KDR*, *SMO*, *HRAS*, *HNF1A*, *RAI1*, and *TP53 *c.430C>G, and the two missense variants of *SND1 *(c.1810G>C and c.1814G>C) were also not reported in COSMIC and ClinVar databases (Table 3). However, an 1814G>T variant had previously been established, causing the substitution of p.(Gly605Val; COSM6786023) in large intestine tissue. Notably, in the HBL tumor of this study, the alteration was p.(Gly605Ala). *ALK *(rs760315884) and *FGFR2 *(rs1042522) missense variants were reported in the SNP database. In addition, only the missense variant p.(Glu707Lys) of *PIK3CA *was reported (COSM5030972) in the ClinVar databases, and in this protein, lysine is substituted in the place of glutamic acid at codon 707. Both are polar AAs; glutamate is negatively charged, and lysine is a positively charged AA. This variant is not present in population databases (ExAC). Results of this study also demonstrated that the allele frequency was low (16.5%) for this mutation (G>A). However, the Poly-Phen-2 score of this variant was 0.998; thus, this variant has been classified as a variant of uncertain significance (VUS). In the same AA codon of *PIK3CA*, a different variant [c.2120A>T; p.(Glu707Val)] was also reported (COSM9644514) in the esophagus. Results of a previous study demonstrated a *KDR *mutation, namely, c.1416A>T; p. (Gln472His) in exon 11, in an anaplastic ependymoma Grade III tumor. In the HBL tumor in this study, c.862T>C; p. (Phe288Leu) was present in exon 7 [20].

The HBL tumor of this study exhibited exon 25 and exon 9 mutations in *ALK *and *FGFR3 *genes (Table 3); however, mutations in exon 29 and exon 16 in these two genes were previously reported in an atypical-choroid plexus papilloma (a-CPP) tumor [21]. In the *ALK *gene, exon 25 contained a c.3750T>G; p. (Ile1250Met) mutation in the HBL tumor in the present study, and another variant (COSM10044698) was reported at this site, c.3748 A>G; p. (Ile1250Val). In the *FGFR3 *gene, another mutation (COSM9495718) in the same AA codon, a c.1246A>G; p. (Ile416Val), was also reported in prostate tissue.

Exon 3 of *CTNNB1 *is a crucial exon coding for several serine-threonine phosphorylation sites, which activates the degradation of β-catenin [31]. Several of the hotspot mutations were reported in exon 3, and the majority of them were found at Ser33, Ser37, Ser45, Thr41, Asp32, and Gly34. Results of a previous study demonstrated a Gly34 mutation in the adamantinomatous craniopharyngioma (ACP) tumor, determined using NGS analysis on Ion Proton [21]. However, in the HBL tumor in the present study, a *CTNNB1 *mutation in c.149 G>T was detected, which had not been previously reported. A c.149 G>A mutation has been shown for substituting Gly50Asp (COSM5718) in four samples, namely, the large intestine, CNS, liver, and hematopoietic samples [32,33]. In the present HBL tumor sample, the G>T mutation caused a Gly50Val substitution (Table 3). In the same AA codon, c.148G>A; p.(Gly50Ser) mutation has also been previously reported (COSM5765). The pathogenic SNP of *TP53*, (rs757274881), c.430C>T; (p. Gln144Ter), was reported in ovarian cancer; however, a different variant, c.430C>G, p. (Gln144Glu), was detected in this codon (Table 3). There are 11 variant types reported in the COSMIC database in c.430; however, variant C>G is a novel variant reported in this study. The *TP53 *c.432 G>T (rs786201419) missense variant was reported in the SNP database as pathogenic, and this c.432G>T; p. (Gln144His) variant was detected in the HBL tumor in this study (Table 3). Notably, this was reported previously in hepatocellular carcinoma and breast neoplasm, and this variant was reported in the COSMIC and ClinVar databases. Other missense mutations also known in this codon (c.432 G>C) caused the substitution Gln144His. In Table 4, the sequencing quality metrics, such as the Phred score, p-value, allele coverage, and allele frequency are presented. There are few low coverage variants in the present tumor sequencing; however, the true mutations have been considered based upon the Phred quality score, i.e., Q = -10log₁₀ P; P is the base calling error probability. That means in a score of 30 to a base, the chances that this base is called incorrectly are 1 in 1,000, and the accuracy is 99.9%. Based upon the high Phred score, and the p-value being less than 0.00001 for most of the variants, we have considered these variants with high confidence in this tumor.

**Table 4:** Quality metrics of missense and INDEL variants found in the hemangioblastoma tumor. cDNA = coding DNA; INDEL = insertion and deletion

Locus	Genes	cDNA	Allele coverage	Allele ratio	P-value	Coverage (x)	Phred quality score
chr1:115258780	NRAS	c.1_2insA	A=17, AT=320	A=0.0504, AT=0.9496	0.00001	337	1216.85
chr2:29432738	ALK	c.3750T>G	A=0, C=399	A=0.0, C=1.0	0.00001	399	3844.05
chr3:41266152	CTNNB1	c.149G>T	G=2, T=67	G=0.029, T=0.971	0.00001	69	615.032
chr3:178938877	PIK3CA	c.2119G>A	G=334, A=66	G=0.835, A=0.165	0.00023	400	36.4671
chr4:1806221	FGFR3	c.1240A>T	A=7, T=5	A=0.5833, T=0.4167	0.00248	12	26.0615
chr4:55979585	KDR	c.862T>C	A=9, G=34	A=0.2093, G=0.7907	0.00001	43	239.4
chr7:127714584	SND1	c.1810G>C	G=0, C=10	G=0.0, C=1.0	0.00001	10	99.2667
chr7:127714588	SND1	c.1814G>C	G=0, C=10	G=0.0, C=1.0	0.00001	10	99.2464
chr7:128845046	SMO	c.540T>G	T=1, G=34	T=0.0286, G=0.9714	0.00001	35	311.833
chr10:123279560	FGFR2	c.872T>G	A=2, C=41	A=0.0465, C=0.9535	0.00001	43	356.767
chr11:534321	HRAS	c.2T>A	A=37, T=16	A=0.6981, T=0.3019	0.00005	53	43.1882
chr12:121431997	HNF1A	c.745delT	AT=21, A=35	AT=0.375, A=0.625	0.00001	56	85.1625
chr17:7578498	TP53	c.432G>T	C=2, A=10	C=0.1667, A=0.8333	0.00001	12	75.9734
chr17:7578500	TP53	c.430C>G	G=1, C=10	G=0.0909, C=0.9091	0.00001	11	84.5839
chr17:17698975	RAI1	c.2713G>A	G=0, A=375	G=0.0, A=1.0	0.00001	375	3549.28

As shown in Table 5, there were 10 synonymous mutations found in the tumor in this study, and one variant was found in each gene, namely, *ALK*, *MLH1*, *FGFR3*, *KIT*, *KDR*, *APC*, *ABL1*, and *RET*, and, in *PDGFRA*, two variants were detected. The synonymous variants found in the tumor in this study, such as *MLH1* (rs769364808), *FGFR3 *(rs7688609), two variants in *PDGFRA *(rs1873778 and rs2228230), *KIT *(rs55986963), APC (rs41115), and *RET* (rs1800861) were reported previously in COSMIC, University of California Santa Cruz (UCSC), SNP, and ClinVar databases. In the tumor presented in this study, the *MLH1 *gene c.1104 G>T mutation was detected, causing a synonymous change at p. (Ser368Ser). At the same AA codon, two other variants are also known to cause missense substitutions, c.1103C>G; [p. (Ser368Trp); COSM6986674)] and c.1103C>T; [p.(Ser368Leu); COSM3915870)], were found in hematopoietic and urinary tract tissue, respectively. However, three synonymous variants found in *ALK*, *KDR*, and *ABL1 *were not reported in any of these databases (Table 5).

**Table 5:** Synonymous mutations found in the hemangioblastoma tumor by NGS on Ion Proton. cDNA = coding DNA; AA = amino acid; NGS = next-generation DNA sequencing

Gene name	Genotype	Reference	Observed allele	Allele frequency (%)	cDNA changed	AA changed	PhyloP
ALK	T/T	A	T	100	c.3753T>A	p. (Ala1251Ala)	0.5
MLH1	T/T	G	T	96.83	c.1104G>T	p. (Ser368Ser)	-1.73
FGFR3	A/A	G	A	100	c.1953G>A	p. (Thr651Thr)	0.06
PDGFRA	G/G	A	G	100	c.1701A>G	p. (Pro566Pro)	-3.59
PDGFRA	C/T	C	T	51	c.2472C>T	p. (Val824Val)	0.64
KIT	A/G	A	G	61	c.1638A>G	p. (Lys546Lys)	1.33
KDR	T/C	T	C	56.52	c.1494A>G	p. (Ile497Ile)	2.89
APC	A/A	G	A	95.5	c.4479G>A	p. (Thr1493Thr)	0.09
ABL1	A/T	A	T	58.97	c.864A>T	p. (Ala288Ala)	-0.23
RET	T/T	G	T	100	c.2307G>T	p. (Leu769Leu)	1.23

In the *ABL1 *gene, a mutation c.864C>T; p. (Ala288Ala; COSM9802919) was detected in the skin, and in the HBL tumor in this study, another mutation in c.864A>T was found that caused the same synonymous change p. (Ala288Ala). Results of a previous study reported intronic *PIK3CA *(rs3729674) and missense *KDR *(rs1870377) variants in anaplastic ependymoma Grade III [20]. Notably, synonymous variants found in this tumor were detected in other tumors, such as *APC *(rs41115) variant in *MPE *[31], *FGFR3 *(rs7688609), *PDGFRA *(rs1873778), and *RET *(rs1800861) in anaplastic ependymoma Grade III tumor, a-CPP, and myxo-papillary ependymoma (MPE) tumors [20,22,23].

Additionally, 19 intronic variants were identified in the present tumor (Table 6). One intronic SNV was found in each of the following genes: *EGFR*, *ERBB4*, *KDR*, *SMO*, *CDKN2B*, *PTEN*, *PTPN11*, *RB1*, *AKT1*, and *ERBB2*. In *PIK3CA *and *FBXL18 *genes, two intronic variants were detected, and in the *SND1 *gene, three intronic variants were detected. Notably, two of the *SND1 *intronic variants (c.1780-50G>A; and c.1780-55C>G) were not reported previously, and only one (c.1780-63 G>A) was reported (COSN28645441; rs225241) in the lung tissue. The intronic mutation of the *AKT1 *gene detected in the tumor of the present study (in c.175+18C>T) was also previously reported in meningioma tumors (COSN17133117). The intronic *TP53 *mutation (c.782+1G>T) found in this study is reported in dbSNP (rs1555525429), COSMIC (COSM44640), and the ClinVar miner database as a pathogenic splice_donor_variant. In the same codon, two other variants were also reported previously c.782+1G>C in the lung and pancreas (COSM329756), and c.782+1G>A (COSM43571) in the breast and lung. These two variants are involved in the alteration of a conserved intronic nucleotide and occur as a somatic driver mutation in patients with a wide variety of cancers, such as osteosarcoma, breast cancer, thymic carcinoma, and small-cell lung carcinoma. These mutations have previously been described in the ClinVar miner database (submission accession SCV000923846). The *TP53 *(c.782+1G>T) variant was not reported in brain tissue so far, and the PolyPhen-2 and SIFT scores predicted that this was likely pathogenic. The intronic variants found in the tumor of the present study were also detected in other tumors; for example, *PIK3CA* (rs3729674) was present in anaplastic ependymoma Grade III and ACP tumors [20,21]. In addition, the *ERBB4* variant (rs839541) was reported in anaplastic ependymoma Grade III [20], and the *KDR* variant c.798+54G>A was reported in anaplastic ependymoma Grade III, ACP, and MPE tumors. In the tumor of the present study, another intronic variant of *KDR*, c.1413-20C>G, was detected [20,21,23]. Intronic variants, such as *AKT1* (rs3730358), *SND1 *(rs225241), *FBXL18 *(rs7799622), *PIK3CA *(rs3729674), and *ERBB4 *(rs839541) were reported in SNP and UCSC databases. Notably, Cold Shock Domain Containing E1 (CSDE1) is an NRAS upstream gene protein (CSDE1) untranslated region (UTR) variant that was not reported in the COSMIC database. Other variants were not reported in any databases. Only the *CSDE1 *gene mutation, (c.*2010T>AT), was a 3'-UTR variant detected in this tumor (Table 6).

**Table 6:** Intronic mutations found in the hemangioblastoma tumor by NGS on Ion Proton. cDNA = coding DNA; NGS = next-generation DNA sequencing

Gene name	Genotype	Reference	Observed allele	Allele frequency (%)	cDNA changed	Transcript	PhyloP
CSDE1	AT/AT	A	AT	94.96	c.*2010T>AT	NM_001242891.1	9.12
ERBB4	T/C	T	C	76.47	c.421+58A>G	NM_005235.2	-3.34
PIK3CA	A/G	A	G	72.22	c.352+40A>G	NM_006218.3	0.78
PIK3CA	A/G	A	G	66.67	c.1252-62A>G	NM_006218.3	2.11
KDR	C/C	G	C	100	c.1413-20C>G	NM_002253.2	0.35
FBXL18	T/T	C	T	97.58	c.1781+161G>A	NM_024963.5	-1.46
FBXL18	C/C	CA	C	100	c.1781+130TG>G	NM_024963.5	-2.13, -1.76
EGFR	G/A	G	A	14.5	c.2283+5G>A	NM_005228.3	9.63
SND1	A/A	G	A	100	c.1780-63G>A	NM_014390.3	-1.41
SND1	C/G	C	G	36.73	c.1780-55C>G	NM_014390.3	-0.24
SND1	G/A	G	A	51.02	c.1780-50G>A	NM_014390.3	0.23
SMO	A/A	G	A	96.3	c.1264+7G>A	NM_005631.4	2.06
CDKN2B, CDKN2B-AS1	A/A	G	A	91.16	c.157-18C>T	NR_047543.1, NM_004936.3	0.73
PTEN	G/T	G	T	81.25	c.79+15G>T	NM_000314.6	0.45
PTPN11	C/C	T	C	100	c.1448-12T>C	NM_002834.3	0
RB1	A/G	A	G	38.46	c.1961-20A>G	NM_000321.2	-0.2
AKT1	G/A	G	A	61	c.175+18C>T	NM_001014431.1	-0.87
TP53	A/A	C	A	100	c.782+1G>T	NM_000546.5	2.22
ERBB2	A/T	A	T	38.33	c.2649+49A>T	NM_004448.3	0

**Figure 12:**
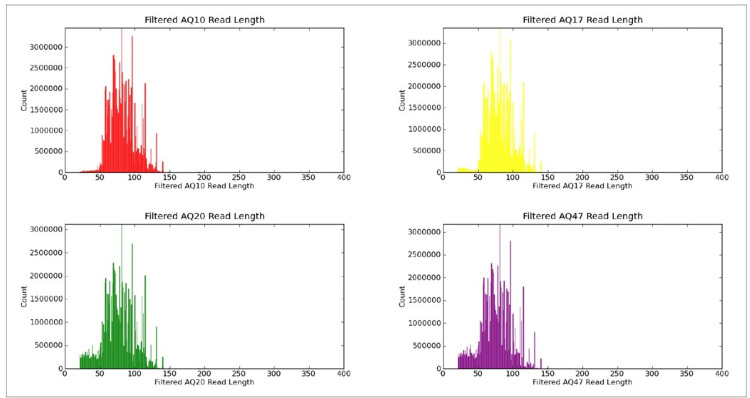
Summary of the filtered reads alignment quality (AQ) to reference genome. (A) Quality of filtered reads AQ10. (B) Quality of filtered reads AQ17. (C) Quality of filtered reads AQ20. (D) Quality of filtered reads AQ47.

However, FATHMM scores, used for the prediction of functional consequences, as described in the COSMIC database, are deleterious above 0.5. Only scores ≥0.7 are classified as pathogenic. Results of the present study demonstrated that the p-value and Phred score was significantly increased in all of the aforementioned mutations. The coverage details of these synonymous and intronic mutations are described in Table 7 and Table 8. Allele ratio, p-value, Phred quality score, sequencing coverage, and allele frequencies are also shown in Table 7 and Table 8. The sequences are of high quality. Figure 12 (Appendices) provides useful sequence information showing AQ10, AQ17, AQ20, and AQ47 of read lengths. AQ20 means one error in 100 base pairs (bp). Any base call with Q<20 was considered low quality, and any variant identified where a substantial proportion of reads supporting the variant are of low quality was considered potentially false positive.

**Table 7:** Quality metrics of synonymous variants found in the hemangioblastoma tumor. cDNA = coding DNA

Locus	Genes	cDNA	Allele coverage	Allele ratio	P-value	Coverage (x)	Phred quality score
chr2:29432735	ALK	c.3753T>A	A=0, T=399	A=0.0, T=1.0	0.00001	399	3825.35
chr3:37067193	MLH1	c.1104G>T	G=2, T=61	G=0.0317, T=0.9683	0.00001	63	545.79
chr4:1807894	FGFR3	c.1953G>A	G=0, A=399	G=0.0, A=1.0	0.00001	399	3825.68
chr4:55141055	PDGFRA	c.1701A>G	A=0, G=400	A=0.0, G=1.0	0.00001	400	3853.33
chr4:55152040	PDGFRA	c.2472C>T	C=196, T=204	C=0.49, T=0.51	0.00001	400	878.619
chr4:55593481	KIT	c.1638A>G	A=156, G=244	A=0.39, G=0.61	0.00001	400	1291.78
chr4:55972896	KDR	c.1494A>G	T=10, C=13	T=0.4348, C=0.5652	0.00001	23	70.3015
chr5:112175770	APC	c.4479G>A	G=18, A=382	G=0.045, A=0.955	0.00001	400	3396.93
chr9:133747557	ABL1	c.864A>T	A=16, T=23	A=0.4103, T=0.5897	0.00001	39	126.316
chr10:43613843	RET	c.2307G>T	G=0, T=396	G=0.0, T=1.0	0.00001	396	3741.83

**Table 8:** Quality metrics of intronic variants found in the hemangioblastoma tumor cDNA = coding DNA

Locus	Genes	cDNA	Allele coverage	Allele ratio	P-value	Coverage (x)	Phred quality score
chr1:115258780	CSDE1	c.*2010T>AT	A=17, AT=320	A=0.0504, AT=0.9496	0.00001	337	1216.85
chr2:212812097	ERBB4	c.421+58A>G	T=4, C=13	T=0.2353, C=0.7647	0.00001	17	91.8018
chr3:178917005	PIK3CA	c.352+40A>G	A=5, G=13	A=0.2778, G=0.7222	0.00001	18	88.4822
chr3:178927912	PIK3CA	c.1252-62A>G	A=4, G=8	A=0.3333, G=0.6667	0.00001	12	52.5318
chr4:55972997	KDR	c.1413-20C>G	G=0, C=396	G=0.0, C=1.0	0.00001	396	3736.44
chr7:5539958	FBXL18	c.1781+161G>A	C=8, T=322	C=0.0242, T=0.9758	0.00001	330	2965.36
chr7:5539989	FBXL18	c.1781+130TG>G	CA=0, C=19	CA=0.0, C=1.0	0.00001	19	113.313
chr7:55242518	EGFR	c.2283+5G>A	G=342, A=58	G=0.855, A=0.145	0.00928	400	20.3268
chr7:127714491	SND1	c.1780-63G>A	G=0, A=49	G=0.0, A=1.0	0.00001	49	471.975
chr7:127714499	SND1	c.1780-55C>G	C=31, G=18	C=0.6327, G=0.3673	0.00001	49	62.6991
chr7:127714504	SND1	c.1780-50G>A	G=24, A=25	G=0.4898, A=0.5102	0.00001	49	117.665
chr7:128846435	SMO	c.1264+7G>A	G=1, A=26	G=0.037, A=0.963	0.00001	27	232.464
chr9:22006264	CDKN2B, CDKN2B-AS1	c.157-18C>T	G=22, A=227	G=0.0884, A=0.9116	0.00001	249	1893.92
chr10:89624320	PTEN	c.79+15G>T	G=3, T=13	G=0.1875, T=0.8125	0.00001	16	95.6742
chr12:112926816	PTPN11	c.1448-12T>C	T=0, C=16	T=0.0, C=1.0	0.00001	16	147.373
chr13:49033804	RB1	c.1961-20A>G	A=8, G=5	A=0.6154, G=0.3846	0.00568	13	22.4564
chr14:105246407	AKT1	c.175+18C>T	G=156, A=244	G=0.39, A=0.61	0.00001	400	1291.67
chr17:7577498	TP53	c.782+1G>T	C=0, A=14	C=0.0, A=1.0	0.00001	14	125.807
chr17:37881506	ERBB2	c.2649+49A>T	A=37, T=23	A=0.6167, T=0.3833	0.00001	60	68.5441

Ion AmpliSeq comprehensive cancer panel (cat. no. 4477685) consists of 16,000 primers supplied in four aliquots and targets 409 genes. The Ion AmpliSeq Cancer HotSpot Panel (cat no. 4471262) consists of 50 oncogenes and tumor suppressor genes that are frequently mutated in several types of cancers. The mutation profiling of a-CPP, MPE, and other brain tumors was reported in previous studies using these custom gene panels on the Ion Proton instrument [20-23,28]. Compared with the Ion AmpliSeq Comprehensive Cancer Panel and Ion AmpliSeq Cancer HotSpot Panel, the custom panel used in the present NGS analysis (Ion AmpliSeq Cancer Panel v1) contains primers for only 46 genes, as shown in Table 1. The *IDH1 *intronic mutation (c.414+9T>A) was previously reported in this HBL tumor by us; however, no missense mutations were detected in *IDH1 *and *IDH2 *genes in this tumor [28]. Ion AmpliSeq™ Cancer Panel v1 contains the primers for the *VHL* gene; however, no *VHL *mutations were detected in this tumor. Previous studies have also reported the absence of *VHL *gene mutations in sporadic cerebellar HBL, consistent with the findings of the present study [34]. However, further studies suggested that mutations in the *VHL *gene are implicated in the development of at least 20% of sporadic CNS-HBL tumors [35]. Several intronic- and exonic-missense and silent mutations were detected in sporadic HBL tumors using NGS in genes such as *ALK*, *KDR*, *CTNNB1*, *PIK3CA*, *FGFR3*, *FGFR2*, *HRAS*, *HNF1A*, *SMO*, and *TP53 *[13]. These genes are present in the custom gene panel used in the present investigation; however, different variants were found in these genes [13]. Ma et al. reported the use of whole-exome sequencing in six sporadic HBL tumors and identified 86 mutations in 67 genes. Several of these variations are known to play essential roles in angiogenesis or the pathogenesis of cancers [35]. The previously reported NGS analysis of CNS-HBL tumors is summarized in Table 9 [13,19,35-37].

**Table 9:** Summary of previously published NGS analysis of central nervous system hemangioblastoma tumors. PGM = personal genome machine; WES = whole-exome sequencing; VHL = von Hippel-Lindau; HBL = hemangioblastoma; NGS = next-generation DNA sequencing

Serial number	Reference	Country	Type of tumors and number of samples	NGS platform used	Primers used	Variants reported
1	Shankar et al. [13]	United States	32 sporadic HBLs of the cerebellum and spinal cord	HiSeq 2500 system	WES of 10 sporadic HBs and targeted sequencing of 560 cancer-associated genes (OncoPanel) in 22 sporadic HBLs	Recurrent loss of chromosome 3
2	Takayanagi et al. [34]	Japan	Eleven VHL-related and 21 sporadic HBLs	Ion Torrent Proton Sequencer	Amplified all exons of VHL	*VHL *mutation/deletion detected
3	Ma et al.e [32]	China	Five familial HBs and six sporadic HBLs	Complete Genomics (CG) platform	WES	Copy number variation; genes harboring the most significant mutations include *PCDH9*, *KLHL12*, *DCAF4L1*, and *VHL *in sporadic HBs, and *ZNF814*, *DLG2*, *RIMS1*, *PNN*, and *MUC7 *in familial HBLs
4	Coppin et al. [19]	France	Sporadic 46 cases (no familial history) and one case was familial (the patient had an affected daughter)	PGM platform, Ion Torrent	Amplified exons 1, 2, and 3 of VHL	VHL mosaic mutations detected
5	Rana et al. [33]	United States	Sporadic HBL (left cerebellar) one case	Illumina HiSeq 2500	OncoPanel	No copy number changes; two VHL variants, p. (Pro81Ser) c.241C>T in exon 1; and p. (Leu63fs*4) c.188_188T>TG

## Conclusions

In summary, we have presented the mutational spectrum of the sporadic, non-VHL-HBL tumor using NGS. The exonic missense variants were detected in multiple genes, including *CTNNB1*, *FGFR3*, *KDR*, *SMO*, *HRAS*, *RAI1*, and *TP53 *variant (c.430C>G). One novel variant in the *TP53 *gene and two missense variants of *SND1 *were also detected. The insertion frameshift variant in *NRAS *and deletion variant in *HNF1A *genes was also novel. The four synonymous variants detected in the HBL tumor of the present study, in genes such as *MLH1*, *ALK*, *KDR*, and *ABL1*, were not reported previously. Among the three intronic variants detected in the *SND1* gene in the HBL tumor, two of which were novel. Only one 3’-UTR variant in the *CSDE1 *gene and an intronic splice_donor_variant of the *TP53 *gene was found in the HBL tumor.

Mutations in other cancer driver genes, such as *PTEN*, *ATM*, *SMAD4*, *SMARCB1*, *STK11*, *NPM1*, *CDKN2A*, and *EGFR*, which are frequently affected in gliomas, were not found in the tumor of this study. Notably, these are rarely reported in HBL tumors, which is in accordance with the NGS analysis of the HBL tumor in this study. Currently, no approved targeted therapy for treating VHL-associated and/or sporadic HBLs exists. As these novel mutations have not been previously reported in HBL tumors, the findings of this study may lead to the development of a genetic signature to distinguish between HBL types, such as supratentorial, VHL, and infratentorial. However, further studies using large samples are required. Future investigations using NGS and animal models may determine additional oncogenic mutations that may act as targets for the treatment of these tumors.

## References

[1] Neumann HP, Eggert HR, Weigel K, Friedburg H, Wiestler OD, Schollmeyer P (1989). Hemangioblastomas of the central nervous system. A 10-year study with special reference to von Hippel-Lindau syndrome. J Neurosurg.

[2] Kuharic M, Jankovic D, Splavski B, Boop FA, Arnautovic KI (2018). Hemangioblastomas of the posterior cranial fossa in adults: demographics, clinical, morphologic, pathologic, surgical features, and outcomes. A systematic review. World Neurosurg.

[3] Hoshide R, Jandial R (2016). 2016 World Health Organization classification of central nervous system tumors: an era of molecular biology. World Neurosurg.

[4] Louis DN, Perry A, Reifenberger G (2016). The 2016 World Health Organization classification of tumors of the central nervous system: a summary. Acta Neuropathol.

[5] Conway JE, Chou D, Clatterbuck RE, Brem H, Long DM, Rigamonti D (2001). Hemangioblastomas of the central nervous system in von Hippel-Lindau syndrome and sporadic disease. Neurosurgery.

[6] Pandey S, Sharma V, Pandey D, Kumar V, Kumar M (2016). Supratentorial haemangioblastoma without von Hippel-Lindau syndrome in an adult: a rare tumor with review of literature. Asian J Neurosurg.

[7] Chang KC, Hsieh CT, Huang JS (2022). Supratentorial hemangioblastoma: a rare case report and literature review. Radiol Case Rep.

[8] Catapano D, Muscarella LA, Guarnieri V, Zelante L, D'Angelo VA, D'Agruma L (2005). Hemangioblastomas of central nervous system: molecular genetic analysis and clinical management. Neurosurgery.

[9] Wong WT, Agrón E, Coleman HR, Tran T, Reed GF, Csaky K, Chew EY (2008). Clinical characterization of retinal capillary hemangioblastomas in a large population of patients with von Hippel-Lindau disease. Ophthalmology.

[10] Lonser RR, Butman JA, Kiringoda R, Song D, Oldfield EH (2009). Pituitary stalk hemangioblastomas in von Hippel-Lindau disease. J Neurosurg.

[11] Mehrian-Shai R, Yalon M, Moshe I (2016). Identification of genomic aberrations in hemangioblastoma by droplet digital PCR and SNP microarray highlights novel candidate genes and pathways for pathogenesis. BMC Genomics.

[12] Lemeta S, Aalto Y, Niemelä M (2002). Recurrent DNA sequence copy losses on chromosomal arm 6q in capillary hemangioblastoma. Cancer Genet Cytogenet.

[13] Shankar GM, Taylor-Weiner A, Lelic N (2014). Sporadic hemangioblastomas are characterized by cryptic VHL inactivation. Acta Neuropathol Commun.

[14] Lui WO, Chen J, Gläsker S (2002). Selective loss of chromosome 11 in pheochromocytomas associated with the VHL syndrome. Oncogene.

[15] Sprenger SH, Gijtenbeek JM, Wesseling P, Sciot R, van Calenbergh F, Lammens M, Jeuken JW (2001). Characteristic chromosomal aberrations in sporadic cerebellar hemangioblastomas revealed by comparative genomic hybridization. J Neurooncol.

[16] Liu Z, Li L, Yi Z (2020). Overexpression of EGFR and TGFα in von Hippel-Lindau-related central nervous system hemangioblastomas. Front Oncol.

[17] Taïeb D, Barlier A, Yang C (2016). Somatic gain-of-function HIF2A mutations in sporadic central nervous system hemangioblastomas. J Neurooncol.

[18] Gagan J, Van Allen EM (2015). Next-generation sequencing to guide cancer therapy. Genome Med.

[19] Coppin L, Plouvier P, Crépin M (2019). Optimization of next-generation sequencing technologies for von Hippel Lindau (VHL) mosaic mutation detection and development of confirmation methods. J Mol Diagn.

[20] Butt E, Alyami S, Nageeti T (2019). Mutation profiling of anaplastic ependymoma grade III by Ion Proton next generation DNA sequencing. F1000Res.

[21] Jastania RA, Saeed M, Al-Khalidi H (2020). Adamantinomatous craniopharyngioma in an adult: a case report with NGS analysis. Int Med Case Rep J.

[22] Taher MM, Hassan AA, Saeed M (2019). Next generation DNA sequencing of atypical choroid plexus papilloma of brain: identification of novel mutations in a female patient by Ion Proton. Oncol Lett.

[23] Taher MM, Alhussini AA, Saeed M (2021). Mutation profiling of intracranial myxopapillary ependymoma by next generation DNA sequencing. Gulf J Oncolog.

[24] Athar M, Al-Allaf FA, Abduljaleel Z, Taher MM, Bouazzaoui A (2022). Design and optimization of 18-gene Ion AmpliSeq panel of Next-generation sequencing for gene mutation analysis causing pain insensitivity. J Umm Al-Qura Univ Med Sci.

[25] Church DM, Schneider VA, Graves T (2011). Modernizing reference genome assemblies. PLoS Biol.

[26] Schneider VA, Graves-Lindsay T, Howe K (2017). Evaluation of GRCh38 and de novo haploid genome assemblies demonstrates the enduring quality of the reference assembly. Genome Res.

[27] Degner JF, Marioni JC, Pai AA, Pickrell JK, Nkadori E, Gilad Y, Pritchard JK (2009). Effect of read-mapping biases on detecting allele-specific expression from RNA-sequencing data. Bioinformatics.

[28] Taher MM, Dairi G, Butt EM (2020). EGFRvIII expression and isocitrate dehydrogenase mutations in patients with glioma. Oncol Lett.

[29] Carney EM, Banerjee P, Ellis CL (2011). PAX2(-)/PAX8(-)/inhibin A(+) immunoprofile in hemangioblastoma: a helpful combination in the differential diagnosis with metastatic clear cell renal cell carcinoma to the central nervous system. Am J Surg Pathol.

[30] Polydorides AD, Rosenblum MK, Edgar MA (2007). Metastatic renal cell carcinoma to hemangioblastoma in von Hippel-Lindau disease. Arch Pathol Lab Med.

[31] Gao C, Wang Y, Broaddus R, Sun L, Xue F, Zhang W (2018). Exon 3 mutations of CTNNB1 drive tumorigenesis: a review. Oncotarget.

[32] Hoshida Y, Hongyo T, Jia X (2003). Analysis of p53, K-ras, c-kit, and beta-catenin gene mutations in sinonasal NK/T cell lymphoma in northeast district of China. Cancer Sci.

[33] Pfaff E, Remke M, Sturm D (2010). TP53 mutation is frequently associated with CTNNB1 mutation or MYCN amplification and is compatible with long-term survival in medulloblastoma. J Clin Oncol.

[34] Kanno H, Kondo K, Ito S (1994). Somatic mutations of the von Hippel-Lindau tumor suppressor gene in sporadic central nervous system hemangioblastomas. Cancer Res.

[35] Ma D, Yang J, Wang Y, Huang X, Du G, Zhou L (2017). Whole exome sequencing identified genetic variations in Chinese hemangioblastoma patients. Am J Med Genet A.

[36] Rana HQ, Koeller DR, Schwartz A (2021). Pathogenicity of VHL variants in families with non-syndromic von Hippel-Lindau phenotypes: an integrated evaluation of germline and somatic genomic results. Eur J Med Genet.

[37] Takayanagi S, Mukasa A, Tanaka S (2017). Differences in genetic and epigenetic alterations between von Hippel-Lindau disease-related and sporadic hemangioblastomas of the central nervous system. Neuro Oncol.

